# Political context is associated with extent of everyday physiological synchrony in older couples

**DOI:** 10.1093/geroni/igaa057.3393

**Published:** 2020-12-16

**Authors:** Theresa Pauly, Karolina Kolodziejczak, Johanna Drewelies, Nilam Ram, Denis Gerstorf, Christiane Hoppmann

**Affiliations:** 1 University of Zurich, Zurich, Switzerland; 2 Humboldt University Berlin, Berlin, Berlin, Germany; 3 Humboldt University Berlin, Berlin, Germany; 4 Stanford University, Stanford, California, United States; 5 University of British Columbia, Vancouver, British Columbia, Canada

## Abstract

Social units such as couples exist within a broader societal and cultural context. Characteristics of this macro-level context may indirectly shape couple dynamics by influencing opportunities and motivation for interdependence, e.g. through legislation and prevalent norms/values. The current study investigates the association between political context (left-right political spectrum) and physiological linkage (cortisol synchrony) in older couples’ daily lives. Older adult couples (N = 162) aged 56 to 89 years (M age = 72.3 years) and residing in Germany provided salivary cortisol samples 7 times daily for a 7-day period. Political context in which dyads lived was quantified with respect to where the federal state of residence was located on the left-right political spectrum using voting data from the 2017 federal election. Links between macro-context and extent of cortisol synchrony were examined using multilevel models, controlling for differences in diurnal rhythm, sex, age, body mass index, and individual-level political orientation. On average, there was evidence of synchrony in fluctuations in partners’ cortisol (b = 0.08, SE = 0.02, p < .001). The extent of cortisol synchrony was moderated by macro-context, such that couples living in a federal state placed further right on the left-right political spectrum exhibited greater cortisol synchrony (b = 0.03, SE = 0.01, p = .010). This new evidence provides a foundation for theorizing about and investigating how specific mechanisms contributing to political context, including family values, gender role attitudes, and laws supporting gender equality contribute to interpersonal linkages of physiological stress responses in daily life.

